# Effect of critical illness on the pharmacokinetics and dose-response relationship of midazolam

**DOI:** 10.1186/cc10937

**Published:** 2012-03-20

**Authors:** D Ovakim, KJ Bosma, GB Young, M Sen, LE Norton, F Priestap, RG Tirona, R Kim, GK Dresser

**Affiliations:** 1 University of Western Ontario, London, Canada

## Introduction

Critically ill patients require sedation to tolerate the interventions necessary to facilitate their care. There is growing evidence, however, that use of sedatives, such as the benzodiazepine midazolam, is associated with delirium and other complications that can lead to prolonged ICU stay and increased mortality. The pharmacokinetics of midazolam in healthy populations has been well characterized, and pharmacodynamic studies demonstrate a predictable dose-response relationship. However, in critical illness, where midazolam is often administered as a continuous infusion, the pharmacokinetic properties are often altered. We sought to investigate whether analysis of midazolam plasma concentrations in combination with electroencephalography (EEG) will better define the effect of critical illness on the pharmacokinetics and clinical response to midazolam, while providing a method to assess the adequacy of sedation thereby minimizing the risks associated with prolonged or over-sedation.

## Methods

For this observational study, patients admitted to the ICU with a diagnosis of sepsis and receiving a continuous infusion of midazolam were screened for inclusion. Upon enrollment, a continuous subhairline EEG was applied and blood samples were collected daily for plasma midazolam quantification. Clinical data and laboratory parameters were followed. Plasma midazolam levels were quantified using liquid chromatography with tandem mass spectroscopy.

## Results

Data were available for nine patients. Midazolam clearance demonstrated wide intersubject variability (range 31 ml/minute to 1,157 ml/minute) although average clearance among all patients (418 ml/minute) was comparable to that of healthy controls. Mean midazolam concentrations for patients with coma were significantly higher than for patients without coma (218 ± 185 ng/ml vs. 106 ± 107 ng/ml). The plasma midazolam concentration inversely correlated with EEG frequency, with maximal slowing in the delta (≤4 Hz) range.

## Conclusion

Midazolam concentrations while on continuous infusion were associated with EEG tracings suggestive of deep sedation. Although clearance was relatively preserved, it varied over a wider range than found in healthy populations. The apparent lower threshold for onset of coma may be a reflection of illness severity, concomitant medication use, and variable clearance during the course of illness. These preliminary results suggest that the combination of continuous bedside EEG and therapeutic drug monitoring may be useful for titrating midazolam infusions and to guide tapering to avoid prolonged coma in patients with variable clearance of midazolam.

